# Editorial: Decoding checkpoint inhibitor-induced endocrinopathies

**DOI:** 10.3389/fendo.2022.987648

**Published:** 2022-07-29

**Authors:** Deborah L. Burnett, Megan B. Barnet, Katherine Samaras

**Affiliations:** ^1^ Immunology Division, Garvan Institute of Medical Research, Darlinghurst, NSW, Australia; ^2^ School of Clinical Medicine, St Vincent’s Healthcare Clinical Campus, Faculty of Medicine and Health, University of New South Wales (UNSW), Sydney, NSW, Australia; ^3^ Department of Medical Oncology, St Vincent’s Hospital, Darlinghurst, NSW, Australia; ^4^ Department of Endocrinology, St Vincent’s Hospital, Darlinghurst, NSW, Australia; ^5^ Clinical Obesity, Nutrition, and Adipose Biology Lab, Clinical Science Pillar, Garvan Institute of Medical Research, Darlinghurst, NSW, Australia

**Keywords:** immune checkpoint (ICP), cancer immunotherapies, irAE, autoimmune disease, endocrinopathies

Immune checkpoint inhibitors have redefined the treatment landscape for many patients with cancer. However, the enhanced responses are often inextricably linked to a new subset of treatment toxicity: immune-related adverse events (irAEs). Although irAEs are often mild, high-grade irAEs still affect many patients, including more than 55% of those receiving combination cytotoxic T-lymphocyte-associated antigen 4 (CTLA-4) and programmed death 1 (PD-1) therapy (1). The clinical presentation of irAEs is diverse, dependent on the organ involved and the precipitating treatment (2). Compared with non-endocrine irAEs, endocrine irAEs are disproportionately associated with sequalae extending beyond treatment cessation and more tightly associated with clinical outcomes (3). Improved understanding of endocrine irAEs may lead to earlier detection before the point of organ failure, tailored preventative and treatment strategies, and greater insight into immune effects of checkpoint inhibition. Diagnosis, management and research into pathophysiology of endocrine-related irAEs represent a unique intersect of specialty knowledge, incorporating immunology, endocrinology, oncology, pharmacology and epidemiology. This Research Topic, Decoding Checkpoint Inhibitor-induced Endocrinopathies, sources multi-disciplinary input to provide a unique depository of articles aimed to help guide clinical practice and stimulate translational research.

Despite the wide scope of this Research Topic, many of the articles present synergistic perspectives on endocrine irAEs. A key theme explored is the distinct nature of autoimmune sequelae induced by different subsets of immune checkpoint inhibitors, and the expected cross-over seen in combination therapy. Muir et al. and Chye et al. highlight the altered relative frequency of common irAEs such as thyroiditis between immune checkpoint inhibitors subclasses, with markedly higher incidence following PD-1 blockade compared with CTLA-4 (3, 4). Wu et al. show a similar trend of PD-1-dominance within checkpoint inhibitor-associated type 1 diabetes mellitus (CIADM), potentially related to particular reliance on the PD-1-axis for pancreatic islet cell tolerance (5). As discussed by Barnabei et al. and Chye et al., several endocrinopathies are more common following CTLA-4-blockade, versus PD-1-blockade, including hypophysitis, while combination treatment leads to greater frequency and severity of irAEs than either agent alone (3, 4). Intriguingly, manifestation of disease can differ even within organ-specific toxicity, as highlighted by the tendency to panhypophysitis in anti-CTLA-4-induced hypophysitis compared with more often isolated ACTH-axis hypophysitis following anti-PD-1 (6–9). This Research Topic extends beyond common immune checkpoint inhibitor therapies with Peris et al. presenting a case report of diabetes mellitus following treatment with alpelisib, a selective phosphatidylinosiyol 3-kinase (PI3K) inhibitor. Together these articles highlight that endocrine irAEs represent a diverse clinical phenotype and that mechanism of checkpoint inhibitor activity (anti-PD-1 versus anti-CTLA-4) determines the observed toxicity profile.

Whilst some novel drug-induced toxicities are clearly defined, such as disturbance of glucose homeostasis following PI3K-inhibition as described by Peris et al., molecular mechanisms leading to most immune-related toxicities are not well understood. Even defining biological drivers of disease remains debated. Wu et al., Chye et al. and Muir et al. present evidence that T cells play a fundamental role in irAE development (10–12). Muir et al. extend this to highlight the role of CD4+ memory activation, and recent findings of T cell receptor diversity significance in response and toxicity (12). There is less consensus around the roles B cells and autoantibodies play in irAE pathophysiology. Chye et al. and Muir et al. show that autoantibodies are common in thyroiditis and Barnabei et al. highlight their prevalence in the rare irAE of central diabetes insipidus (13, 14). Studies outlining the correlation of pre-treatment anti-thyroid antibodies with thyroid-related irAEs support a B-cell related predisposition (15). Similarly, Wu et al. cited pre-treatment serum samples from three of six patients who developed CIADM contained classic type 1 diabetes islet autoantibodies, with two patients seroconverting to islet autoantibody positivity after immune checkpoint inhibitor treatment (16–18).

Another key theme addressed within these articles is the contribution of host predisposition to irAEs and the shared genomic drivers of primary and immune checkpoint inhibitor-induced autoimmunity. IrAEs likely result from a complex interplay of host, drug and environment. Genetic predisposition of the host to irAEs is one area of growing interest, particularly given the potential for shared drivers of toxicity and anti-cancer response. Wu et al. highlight that the genetic predisposition for type 1 diabetes mellitus is well established, with HLA polymorphisms in particular playing a dominant role in establishing predisposition to this irAE (19–21). There is mixed evidence, however, regarding the role of type 1 diabetes HLA susceptibility haplotypes in development of CIADM. Muir et al. and Chye et al. present stronger evidence for shared genetic predisposition to organic and immune checkpoint inhibitor-induced thyroid disease. This association was underpinned by a large GWAS-based study showing a relationship between a higher polygenic risk score for hypothyroidism and risk of immune checkpoint inhibitor-induced thyroiditis, and improved cancer outcomes in select groups (22). As highlighted by Barnabei et al., Muir et al. and Chye et al., there are many single nucleotide polymorphisms (SNPs) with shared risk for endocrine irAEs and primary autoimmunity (22–24). Variable weight of effect, however, and often high population prevalence make these hard targets for predictive biomarkers. Interestingly all three of these papers highlight the role of CTLA-4 polymorphisms in predisposition to irAEs, particularly following PD-1 blockade (22, 25, 26). This is a logical follow-on to the observed increase in toxicity seen with combined pharmacological blockade of CTLA-4 and PD-1 (1), and supports the hypothesis that genetic blockade may have some equivalence to pharmacologic blockade.

Together the articles in this Research Topic form a critical contribution to the knowledge base for immune checkpoint inhibitor-induced endocrine irAEs. The articles cover a wide scope of endocrine irAEs, providing both molecular insights and practical guidance for diagnosis and management. Experts within the field explore aetiology and pathology of endocrine events both common and rare, providing an invaluable resource, particularly in areas with narrow existing literature base. Finally, this Research Topic explores the many parallels of endocrine irAEs with organic endocrinopathies. In forging pathways for greater understanding of immune checkpoint inhibitor-induced disease, this Research Topic invites further research into tailored treatment options and potential predictive biomarkers that might improve patient care.

## Author contributions

All authors listed have made a substantial, direct, and intellectual contribution to the work and approved it for publication.

## Funding

This work was supported by National Health and Medical Research Council (NHMRC) (Grants 10375), and by Bioplatforms Australia and The Kinghorn Foundation as part of the Australian Exceptional Responders Program (Grants PR10285).

## Conflict of interest

The authors declare that the research was conducted in the absence of any commercial or financial relationships that could be construed as a potential conflict of interest.

## Publisher’s note

All claims expressed in this article are solely those of the authors and do not necessarily represent those of their affiliated organizations, or those of the publisher, the editors and the reviewers. Any product that may be evaluated in this article, or claim that may be made by its manufacturer, is not guaranteed or endorsed by the publisher.
